# Development and evaluation of a multiplex molecular point-of-care assay for direct identification of *Mycobacterium tuberculosis* and prioritized non-tuberculous mycobacteria

**DOI:** 10.3389/fcimb.2025.1560870

**Published:** 2025-08-13

**Authors:** Qiao-Lian Yi, Yun Wu, Shuang He, Meng-Li Feng, Xiao-Yu Liu, Xin-Zhu Zhou, Hao-Tian Gao, Yu-Fan Zhang, Qi-Wen Yang, Ying-Chun Xu

**Affiliations:** ^1^ Department of Laboratory Medicine, Peking Union Medical College Hospital, Chinese Academy of Medical Sciences and Peking Union Medical College, Beijing, China; ^2^ Graduate School, Chinese Academy of Medical Sciences and Peking Union Medical College, Beijing, China; ^3^ R&D Department Genewise Bio Co., Ltd, Beijing, China; ^4^ State Key Laboratory of Complex, Severe, and Rare Diseases, Chinese Academy of Medical Sciences and Peking Union Medical College, Beijing, China

**Keywords:** non-tuberculous mycobacteria, *Mycobacterium tuberculosis*, pathogen identification, point-of-care testing, molecular diagnosis

## Abstract

**Objective:**

This study aimed to establish a multiplex molecular point-of-care assay called *fastNTM* incorporating an ultra-fast sample pre-treatment for direct identification of *Mycobacterium tuberculosis* complex (MTBC) and 8 non-tuberculous Mycobacteria (NTM) commonly prioritized in clinical settings, and to evaluate its performance in 149 clinical confirmed mycobacterial-positive samples.

**Methods:**

The study was divided into two stages: a pilot study to establish the methodology and a clinical validation study to evaluate its performance. In the pilot study, we established the *fastNTM* and analyzed its performance regarding limits of detection, reproducibility, specificity and efficiency. The clinical validation study was performed using 149 clinical confirmed mycobacterial-positive samples, with 16S rRNA identification as the reference standard. The complete process, from patient to result, was accomplished within 90 minutes.

**Results:**

Of the 149 positive clinical mycobacterial cultures analyzed, 136 were within the designed targets. Among these 136 cultures, 133 samples were correctly identified by *fastNTM*, achieving an accuracy rate of 97.79%.

**Conclusions:**

This study demonstrates that *fastNTM* with its high accuracy rate are capable to rapidly and effectively differentiate between MTBC and the major NTM species.

## Introduction

Non-tuberculous mycobacteria (NTM) represent a diverse group of near 200 species, distinct from the *Mycobacterium tuberculosis* complex (MTBC) and *Mycobacterium leprae* (LPSN, 2025). Recognized as opportunistic pathogens, NTM are more likely to colonize the respiratory tracts of hosts. The global significance of NTM diseases has been escalating due to a rising trend in their occurrence (Kumar and Loebinger, 2022; Cristancho-Rojas et al., 2024). Pulmonary infections caused by NTM present symptoms similar to those of tuberculosis (TB), including persistent cough, weight loss, and fatigue (Dahl et al., 2022). Misdiagnosing NTM infections as recurrent TB or drug-resistant TB often leads to inappropriate empiric treatment, potentially contributing to the development of TB drug resistance (Gopalaswamy et al., 2020). Therefore, rapidly distinguishing between MTBC and NTM, and identifying the specific NTM species, is crucial for the implementation of TB control strategies and patient clinical outcomes.

The diversity of NTM isolated from pulmonary samples varies widely by geographical region. Globally, about one-half of NTM species isolated from human specimens belongs to the M. avium complex (MAC) (Hoefsloot et al., 2013). However, in China, *M. abscessus* and the *M. avium* complex account for up to 52.6% and 23.2% of all NTM-caused infections, respectively (Tan et al., 2021). Despite these regional differences, *M. avium*, *M. abscessus*, *M. fortuitum* and *M. kansasii* remain the most relevant species for NTM pulmonary disease (Kim et al., 2019; Ratnatunga et al., 2020; Dahl et al., 2022; Nguyen et al., 2024). *M. avium* and *M. intracellulare* have higher infection rates among non-AIDS patients (Pasipanodya et al., 2017). Other species, such as *M. scrofulaceum*, *M. gordonae*, *M. marinum* and *M. ulcerans*, although relatively rare in pulmonary infections, have shown a relatively high isolation rate in clinical settings. These species can cause infections not only in the immunocompromised hosts, but also in otherwise healthy individuals (Van der Werf et al., 1999; Chang et al., 2021; Canetti et al., 2022). Pulmonary *M. scrofulaceum* disease has mainly occurred in patients with chronic pulmonary diseases (Suzuki et al., 2016; Wilson et al., 2019).

The conventional methods for identifying NTM in clinical settings, which rely on smear microscopy and culture. Sputum culture, while sensitive and considered the gold standard, is laborious and time-consuming, with a requirement of 10–42 days for results (Forbes et al., 2018). Clinical practices for rapid identification, such as Gene Xpert, are primarily focus on detecting MTBC with limited capacity to identify NTM (Opota et al., 2016). Other reported available assays for the direct identification of NTM in clinical specimens, including the LightCycler Mycobacterium Detection Kit (Omar et al., 2011), Genoscholar™ NTM+MDRTB II (Mitarai et al., 2012), and the GenoType Mycobacteria Direct test (Franco-Álvarez De Luna et al., 2006), still necessitate additional manual operations, sample processing and nucleic acid extraction. Advanced methods such as targeted next-generation sequencing (tNGS) and metagenomic next-generation sequencing (mNGS) can detect a broader range of strains and minimize the risk of missed detections (Schwab et al., 2024). However, limitations of these sequencing-based detection methods are obvious, including the need for costly sequencing instruments, and skilled personnel for high-quality nucleic acid extraction. Otherwise, Mass spectrometry relies on databases for identification. Current databases may not have comprehensive entries for all mycobacterial species, leading to potential misidentification, especially for rare or closely related species (Alcaide et al., 2018). Rapid testing options, while offering faster results, are often limited in the breadth of detectable species or involve complex operational steps.

For species identification, the intergenic transcribed spacer (ITS) region, located between the 16S rRNA and 23S rRNA genes, is a highly conserved yet variable genetic region that has emerged as a crucial target for the detection and identification of genotypically similar bacteria such as non-tuberculous mycobacteria (NTM). The presence of unique sequences within the ITS region allows for the design of highly specific primers and probes, reducing the likelihood of cross-reactivity with other bacterial species (Ngan et al., 2011). To address these challenges, we developed a multiplex PCR-based point-of-care assay called *fastNTM* for MTBC and NTM discrimination along with an ultrafast sample pretreatment method. Given the diverse range of NTM species and their various distribution, eight prevalent NTM species were selected, including *M. avium*, *M. abscessus*, *M. fortuitum*, *M. kansasii*, *M. intracellulare*, *M. scrofulaceum*, *M. gordonae*, and *M. marinum/ulcerans*.

The objective of this study was to evaluate the clinical performance of this panel in differentiating MTBC and key NTM based on multiplex molecular POCT and ultrafast sample pretreatment. By offering a “sample-in, result-out” solution, *fastNTM* represents a significant advancement in the rapid and accurate diagnosis of TB and NTM infections, potentially reducing the time to treatment and enhancing patient outcomes.

## Materials and methods

### Primer and probe design

The assay enables the simultaneous detection of 9 targets in a single reaction, allowing for the concurrent identification of the *M. tuberculosis* complex and 8 NTM. It was achieved by multiplex quantitative PCR platform combined with asymmetric PCR and multicolor melting curve analysis (MMCA) (Elenitoba-Johnson et al., 2001). It targets the intergenic transcribed space (ITS) region between the 16S rRNA and 23S rRNA genes of mycobacteria, using a pan-mycobacterial primer set: By downloading over 20 sequences for each species from the NCBI database, conserved regions were identified through sequence alignment, while overlapping regions with other species were excluded. Specific primers for each species were designed to target theses specific regions using Oligo7 and NCBI BLAST. The NCBI Primer-BLAST was used to verify optimized oligonucleotide sequences for specificity. The species-specific primers exhibited varying melting temperatures and the sequences of the primers-probe pairs are presented in **Supporting Information Table S1** (**Supplementary Material**).

### Establishment of *fastNTM*

**Figure 1:**
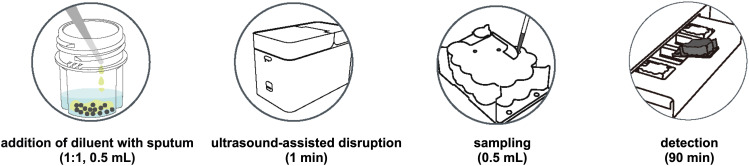
The overall operation procedure of *fastNTM*: Step1. Transfer 0.5 mL of specimen to the pretreatment cartridge with the equal volume of pretreatment solution. 2. Turn on the ultrasound-assisted disruption for 1 minute. 3. Aspirate 0.5 mL of the liquefied sample to the panel. 4. Multiplex PCR will be performed within 85–90 minutes.

The PCR amplification system is carefully calibrated with primer concentrations of 0.6 μM for the *M. tuberculosis* complex and 0.2 μM for restrictive NTM primers, along with 4 μM for non-restrictive primers, ensuring specific detection. The probe concentration is set at 0.1 μM to facilitate the identification of amplified products through fluorescence during the reaction. The multiplex molecular identification is performed in a cartridge. The cartridge comes preloaded with magnetic beads, lysis solution, wash solution, elution buffer, and freeze-dried detection reagents, integrating nucleic acid extraction, RT-PCR amplification, and target detection (**Figure 1**).

### Operation procedure of multiplex molecular point-of-care panel

The overall operation procedure of *fastNTM* is illustrated in **Figure 1**. Initially, 0.5 mL of the original clinical specimen, such as sputum, is transferred to the pretreatment cartridge, mixed with an equal volume of pretreatment solution. The ultrasound-assisted disruption is then activated for 1 minute to homogenize the sample. Following this, 0.5 mL of the liquefied sample is aspirated into the detection cartridge. The analysis is subsequently performed automatically by the NAT - 3000 Pro system (Genewise Bio Co., Ltd, Beijing, China), which completes the process within 85–90 minutes. MTBC determination is characterized by cycle threshold (Ct) value and that of NTM is melting temperature (Tm). The total turnaround time from sample to result is within 90 minutes.

### Sample preparation

**Low, medium, and high concentration tests.** Inactivated *M. tuberculosis* was added to mycobacterial-negative sputum samples at low (40 CFU/mL), medium (1000 CFU/mL), and high concentrations (1,000,000 CFU/mL). For each concentration, 20 replicate tests were conducted for both the pretreated and untreated samples to compare the results.
**Limit of detection (LOD) tests.** The LoD was determined using serial dilutions of MTBC and NTM species. For each of the eight NTM species, concentrations of 100, 300, 500, 750 and 1000 CFU/mL were prepared. While for MTBC, concentrations of 10, 30, 50, 75, and 100 CFU/mL inactivated *M. tuberculosis* were prepared. Those bacterial suspensions were added to mycobacterial-negative sputum samples. Ten replicates were prepared for each species. Mycobacterial-negative sputum samples without additional mycobacterial templates served as negative controls. In each experimental group, both Ct and Tm values included internal controls (IC).
**Comparison with Xpert MTB/RIF Ultra assay.** TB-suspected sputum samples were collected from clinical settings, and different levels of MTB are confirmed by Xpert MTB/RIF Ultra Assay, including 15 MTB not detected, 1 MTB detected very low, 6 MTB detected low, and 8 MTB detected medium. Each sputum sample was divided into two aliquots: one was processed according to the manufacturer’s instructions for the Xpert MTB/RIF Ultra Assay and tested using the GeneXpert^®^ System, while the other was processed according to the *fastNTM* operation procedure as shown in **Figure 1**.

### Clinical mycobacterial cultures preparation

A total of 149 clinical mycobacterial cultures in mycobacteria growth indicator tube (MGIT) originally cultured from 142 respiratory (sputum, tracheobronchial aspirate, bronchoalveolar lavage fluid, endotracheal aspirate) and 7 non-respiratory routine samples (tissue, lymphaden, body fluid) were randomly collected in the Clinical Microbiology Laboratory, Peking Union Medical College Hospital in Beijing, China, between 2021 and 2023. The clinical information of original specimens was shown in **Table 1**. All cultures were frozen samples accumulated over the years, and positive for MGIT system (BD BACTEC MGIT960 system, Becton Dickinson, Franklin Lakes, NJ, USA). Clinical workflow of positive mycobacterial cultures confirmation was presented in **Supporting Information Figure S1** (**Supplementary Material**).

**Table 1:** Clinical information of 149 mycobacterial cultures.

Issues	Number (*n*)	Percentage(%)
Gender, female	99	66.44%
Age distribution		
0-20	4	2.68%
21-40	24	16.11%
41-60	54	36.24%
61-80	56	37.58%
≥81	11	7.38%
Sample type		
Sputum	109	73.15%
Tracheobronchial aspirates	22	14.77%
Bronchoalveolar lavage fluid	9	6.04%
Skin and tissue	3	2.01%
Tracheal intubation aspirates	2	1.34%
Vertebral puncture tissue	1	0.67%
Node biopsy	1	0.67%
Other respiratory specimens	1	0.67%
Other body fluids	1	0.67%
Department Distribution		
Department of Infection	72	48.32%
Respiratory Medicine Clinic	47	31.54%
Respiratory medical wards	20	13.42%
Department of cardiology	10	6.71%

Each clinical mycobacterial MGIT-positive culture was divided into two portions for further analysis. One portion was directly tested with the multiplex molecular POCT panel, while the other underwent nucleic acid extraction and then being sent for 16S rRNA sequencing to obtain species-level identification. Results of 16S rRNA identification were regarded as the reference standard. For strains that cannot be identified by 16S rRNA, further identification is performed using the *hsp*65 gene.

### Statistical analyses

We compared the rates of positive results of both *fastNTM* and reference methods using Cohen’s Kappa Statistic. The 95% confidence intervals (95% CI) were calculated using the Wilson score method. Cohen’s Kappa values for quantify agreement were calculated using GraphPad QuickCalcs (https://www.graphpad.com/quickcalcs/kappa1/) with 95% CIs.

## Results

### Performance of ultrafast pretreatment procedure

**Figure 2:**
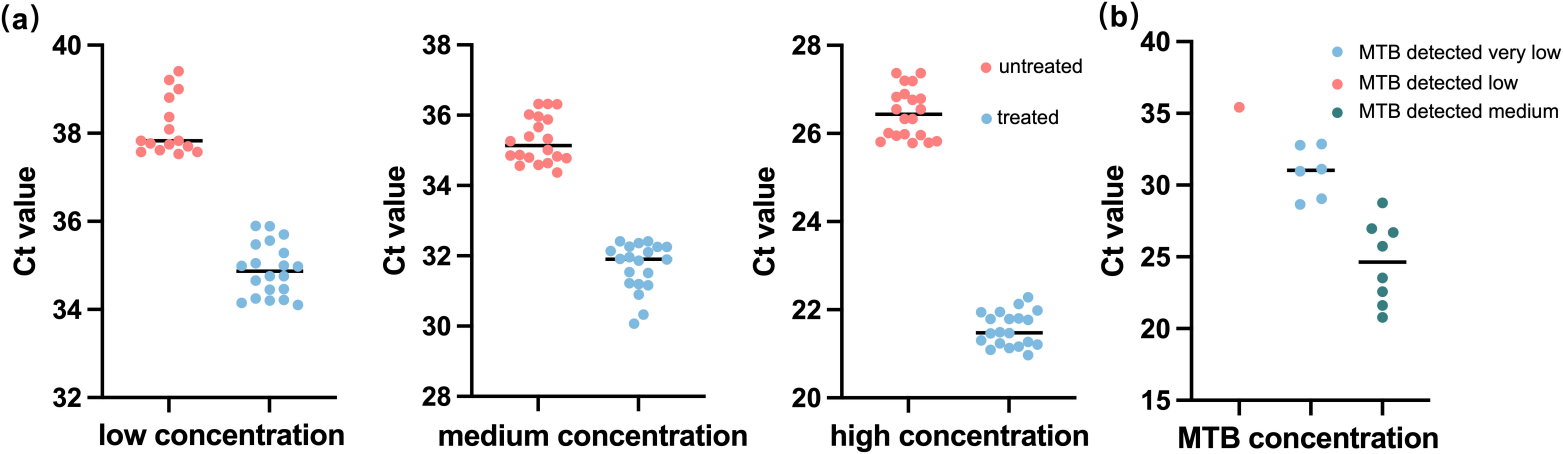
Performance of the ultrafast sample pretreatment procedure in sample detection. **(a)** Comparison results between *fastNTM* with and without the ultrafast sample pretreatment procedure among low (40 CFU/mL), medium (1000 CFU/mL), and high concentrations (1,000,000 CFU/mL). **(b)** Detection results of *fastNTM* using the ultrafast sample pretreatment procedure with the Xpert MTB/RIF Ultra Assay TB-confirmed sputum sample.

An ultrafast sample pretreatment procedure utilizing ultrasound-assisted enzymatic homogenization was implemented in the *fastNTM* assay. To evaluate the impact of this pretreatment method on detection outcomes, a controlled experiment was conducted using simulated samples with low, medium, and high concentrations of inactivated *M. tuberculosis*. The findings are presented in **Figure 2a**. The treated samples displayed cycle threshold (Ct) values of (21.561 ± 0.386, 31.687 ± 0.684, 34.892 ± 0.592) for the high, medium, and low concentration groups, respectively. Conversely, the untreated samples exhibited Ct values of 26.464 ± 0.562, 35.288 ± 0.653, and 38.122 ± 0.652 for the corresponding concentration groups. Within each group, a significant reduction in Ct values was observed, with the low concentration group showing that four samples did not yield detectable nucleic acid levels without treatment. This pretreatment process enhanced the efficiency of nucleic acid release.

### Limit of detection for *fastNTM*

The lowest concentration level that achieved a 100% positive detection rate was determined as the LOD, and this was verified. The final LOD for the *fastNTM* was determined to be 500 copies/mL for *M. tuberculosis* and 500 copies/mL for each NTM species. The coefficients of variation (CV) for the Ct values of *M. tuberculosis* and the Tm values of the eight NTM species were calculated, as well as the IC. The percentage of positive assays for the MTB and 8 NTM in the LoD groups was 100%. The CV for the Tm values of the eight NTM species was less than 1%, the CV for the Ct values of M. tuberculosis was 1.14%, the CV for the IC Ct values was less than 1.8%, and the CV for the IC Tm values was less than 1% (**Supplementary Material**, **Supporting Information Table S2**). The negative samples showed no amplification.

### Comparison of *fastNTM* to GeneXpert

To demonstrate that a 1-minute pretreatment process can achieve the same detection efficiency as a conventional 20-minute procedure, we conducted a comparative analysis using sputum specimens tested with the GeneXpert system. As there are currently no similar products available for *fastNTM*, we have relied solely on the detection results for *M. tuberculosis* as a reference. The comparative results between the *fastNTM* assay and the Xpert MTB/RIF Ultra Assay are presented in **Figure 2b**. It showed a high level of concordance in terms of both negative and positive results. Additionally, the Ct value distribution for *M. tuberculosis* detected by the *fastNTM* assay is consistent with the concentration trends obtained by the Xpert MTB/RIF Ultra Assay, suggesting a strong correlation between the two methods, indicating that the 1-minute pretreatment process can achieve the same detection efficiency as a conventional 20-minute procedure.

### Multiplex molecular POCT analysis of clinical mycobacterial cultures

Of the 149 clinical mycobacterial cultures analyzed in the study, 15 mycobacterial species were identified by 16S rRNA sequencing. The results of *fastNTM* evaluation are presented in **Tables 2**, **3**. A total of 145 cultures were positive detected by *fastNTM*, and 16 cultures showed discrepancies with the established identification results. Among these, cultures of *M. lentiflavum* and *M. shinjukuense* were not detected by *fastNTM*. Cultures of *M. malmoense* and *M. paragordonae* were mis-identified as *M. fortuitum* and *M. gordonae*, respectively. Cultures of *M. colombiense* and *M. marseillense* were identified as *M. intracellulare*. Additionally, one culture of *M. kansasii* were not detected by *fastNTM*, and 2 *M. intracellulare* were identified as *M. avium* (**Table 2**).

**Table 2:** Identification of clinical mycobacterial cultures.

16S rRNA-identified Results	Number of cultures	Number of *fastNTM*-matched cultures	Description of mismatches
*M. intracellulare*	61	59	2 identified as *M. avium* by *fastNTM*
*M. abscessus*	34	34	
*M. avium*	12	12	
*M. fortuitum*	11	11	
*M. gordonae*	7	7	
*M. kansasii*	6	5	1 undetected by *fastNTM*
*M. mageritense*	6	0	identified as *M. fortuitum*, not the target of *fastNTM*
*M. paragordonae*	2	0	identified as *M. gordonae*, not the target of *fastNTM*
*M. lentiflavum*	2	0	negative result, not the target of *fastNTM*
*M. intracellulare* & *M. gordonae*	1	1	
*M. intracellulare* & *M. abscessus*	1	1	
*M. marinum/ulcerans*	1	1	
*M. tuberculosis*	1	1	
*M. scrofulaceum*	1	1	
*M. colombiense*	1	0	identified as *M. intracellulare*, not the target of *fastNTM*
*M. marseillense*	1	0	identified as *M. intracellulare*, not the target of *fastNTM*
*M. shinjukuense*	1	0	negative result, not the target of *fastNTM*

**Table 3:** Statistical results of *fastNTM* compared with reference results.

Species	True positive	False positive	False negative	True negative	Kappa	SE of kappa	95% CI	sensitivity	specificity
*M. intracellulare*	61	2	2	84	0.945	0.027	0.892-0.998	0.9683	0.9767
*M. abscessus*	35	0	0	114	1	0	1.0-1.0	1	1
*M. avium*	12	2	0	135	0.916	0.059	0.8-1.0	1	0.9854
*M. fortuitum*	11	6	0	132	0.765	0.091	0.585-0.944	1	0.9565
*M. gordonae*	8	2	0	139	0.882	0.082	0.72-1.0	1	0.9858
*M. kansasii*	5	0	1	143	0.906	0.904	0.722-1.0	0.8333	1
*M. marinum/ulcerans*	1	0	0	148	1	0	1.0-1.0	1	1
*M. tuberculosis*	1	0	0	148	1	0	1.0-1.0	1	1
*M. scrofulaceum*	1	0	0	148	1	0	1.0-1.0	1	1

*SE: standard error.

Among the 149 cultures, 2 were identified for multiple mycobacterial species by *fastNTM*. Thus, subcultures were performed to obtain single colony. A follow-up 16S rRNA sequencing were identified individually for those single species. The identification results for these sub-cultures showed consistent with *fastNTM*.

Results of 136 cultures confirmed by 16S rRNA identification were within the detection range of this panel, account for 91.28%. Among these 136 cultures, *fastNTM* results of 133 cultures showed concordant identification with the sequencing data, achieving an overall accuracy rate of 97.79%. The sensitivity and specificity of *fastNTM* for each targeted species were showed in **Table 3**. The specificities of all 9 targets were above 95%. Except for *M. kansasii*, the remaining 8 targets showed sensitivities above 96%.

## Discussion

Here we introduced a multiplex molecular point-of-care panel that utilizes one minute of sample processing to simultaneously detect the presence of eight clinically dominant NTM species and MTBC was developed. This method allows for the amplification of multiple targets in a single reaction by using differentially labeled probes for each target. Asymmetric PCR ensures that one strand of the target DNA is preferentially amplified, which enhances the efficiency of probe hybridization. The MMCA then allows for the differentiation of these targets based on their unique melting temperatures. The assay employs an ultrafast sample pretreatment method, reducing the conventional 20-minute process, and achieves automated PCR detection and result analysis within 90 minutes from patient to result. This streamlined protocol achieved an overall accuracy rate of 97.79%.

In this study, the selection of eight prevalent NTM species was primarily based on strain isolation rates (Huang et al., 2020) and epidemiological investigations. Our selection took into account both isolation frequency and clinical pathogenicity. However, this approach may have led to the omission of some clinically significant but less prevalent NTM species, or the inclusion of those with high isolation rates but lower clinical relevance. Our goal is to quickly provide the most probable TB/NTM results to clinicians. This aids in rapid screening and diagnosis, particularly for slowly growing NTM. Among these, *M. marinum* and *M. ulcerans* with high phylogenetic relatedness (i.e., >99.8% 16S rRNA sequence similarity) (Röltgen et al., 2012) were not differentiated in this study. While infections due to *M. marinum* can usually be treated with antimycobacterial drugs, very few cases of *M. ulcerans* infection respond favorably to antimicrobial therapy (Franco-Paredes et al., 2018). One of limitations of this study is that, differentiation of these two species needs further test such as whole-genome method.

In this study we utilized ITS as the target for MTBC and NTM detection. However, when it comes to species-level identification of more NTMs, the ITS region does have certain limitations. For instance, five samples initially identified as *M. fortuitum* by *fastNTM* were later confirmed to be *M. mageritense* through *hsp65* gene sequencing. To achieve more precise differentiation, additional genes such as *hsp65*, *sodA*, *recA*, and *rpoB* are recommended for NTM identification (Adékambi and Drancourt, 2004). Moving forward, if our research involves the identification of a broader range of NTM species, we will incorporate more genes into our primer design to enhance the accuracy and specificity of our detection method.

All nine detection targets in our study demonstrated specificities above 95%, indicating a high level of accuracy in identifying the targeted mycobacterial species. Except for *M. kansasii*, the remaining eight targets showed sensitivities above 96%, which is a significant benchmark for effective diagnostic testing. However, it is important to note that the sample sizes for *M. kansasii*, *M. marinum*/*ulcerans* and *M. scrofulaceum* were less than ten, which could potentially impact the statistical confidence of the sensitivity analysis for these specific targets. This consideration is crucial as smaller sample sizes can lead to less precise estimates of sensitivity and specificity, and may not fully capture the performance characteristics of the detection targets. Therefore, while the high specificity and sensitivity rates are promising, further testing with larger sample sizes for these four targets is necessary to confirm the observed performance metrics.

Besides, among all misidentifications observed in the study, several non-targeted mycobacterial species were incorrectly identified. Specifically, six strains of *M. mageritense*, not within the target range of our assay, were incorrectly identified as *M. fortuitum*. *M. mageritense* was first discovered in 1997 and further study showed its phenotypic and clinical similarity to isolates of the *M. fortuitum* third biovariant complex (sorbitol positive) (Wallace et al., 2002). Without specific method, isolates of *M. mageritense* are likely to go undetected. Two strains of *M. paragordonae* were identified as *M. gordonae*. *M. paragordonae* is an emerging pathogen in human pulmonary disease, first discovered in 2014 (Li et al., 2022). The phylogenetic tree of the 16S rRNA gene sequences showed that the *M. paragordonae* isolates were most closely related to the *M. gordonae* ATCC 14470 T strain, with up to a 99.0% gene match; however, the DNA–DNA affinity comparison between those two isolates was only 46.52%. One strain each of *M. marseillense* and *M. colombiense* were identified as *M. avium*. Both species belong to the *M. avium* complex (MAC). *M. marseillense* was firstly described as a new species within the MAC in 2009 (Salah et al., 2009). One strain of *M. shinjukuense* and two strains of *M. lentiflavum*, which do not belong to MAC, were identified as negative. These misidentifications underscore the complexities in differentiating between closely related mycobacterial species and the necessity for continuous refinement of diagnostic assays to enhance specificity and inclusivity.

To date, in our country, there are three certified commercially available kits for NTM species identification, including the CapitalBio Mycobacterium identification array assay (CapitalBio Corp., Beijing), PCR-REBA Myco-ID assay (Yaneng BioSciences, Shenzhen), and the MeltPro Myco assay (Zeesan Biotech, Xiamen). While these kits offer advantages in terms of the number of species they can identify, they are limited by complex manual operations and varying clinical relevance. CapitalBio Mycobacterium identification array assay uses DNA microarray technology to identify 17 species of mycobacteria. However, it requires samples to be cultured and confirmed as mycobacterial positives before further species identification (Liu et al., 2012). PCR-REBA Myco-ID assay, based on 16S rRNA sequencing and nucleic acid probes with reverse blot hybridization, can identify 22 species of mycobacteria (Zhang et al., 2021). The entire detection process requires a total of 6 hours. Those two kits are more laborious compared to PCR-based methods due to the complex manual operations involved in DNA hybridization.

MeltPro Myco Assay uses multicolor melting curve analysis to identify 19 clinically relevant mycobacterial species. While it offers a higher number of identifiable species (19 vs. 9) and a lower limit of detection (300 CFU/mL vs. 500 CFU/mL), it still requires extensive sample preparation. The patient’s sputum must undergo decontamination, neutralization, centrifugation, resuspension, and DNA extraction before the final DNA detection. The turnaround time for this qPCR-based method is within 3 hours, compared to our 90-minute process (Xu et al., 2019). Besides, the qPCR instrument requires the capability to perform PCR-high resolution melting (PCR - HRM) analysis. Our fastNTM assay addresses these limitations by offering a streamlined, rapid, and clinically relevant detection method. It enables direct detection of TB and NTM from raw patient samples within 90 minutes, significantly improving the speed and reliability of mycobacterial identification in clinical settings.

In this study, among the 149 mycobacterial-positive cultures, 43 (28.86%; 43/149) showed negative smear results despite being positive in the MGIT system. According to our hospital’s protocol, further sub-culture on Löwenstein-Jensen medium was performed for these MGIT960-positive cultures. This means that for these 43 samples, there are an average delay of 15.59 ± 10.38 days in confirming NTM positivity for these samples, with a median delay of 12.5 days and the longest extension reaching 41 days after MGIT960 positive-alert. Notably, all 43 of these cultures were detected by *fastNTM*, without the need for further cultivation. Excluding four samples that were outside the target range, f*astNTM* achieved a 100% accuracy rate in detecting these cultures (**Supplementary Material**, Information of NTM.xlsx). This highlights the superior sensitivity and efficiency of f*astNTM* in identifying NTM-positive samples compared to traditional smear methods.

Additionally, the ultrafast sample pretreatment procedure introduced here that requires only one minute, significantly enhancing overall detection efficacy. For instance, in our tests, the LoD for MTBC is 50 copies. However, in pure bacterial suspensions without a negative sputum matrix, a concentration of 40 copies can achieve 100% detection after undergoing the sample pretreatment. In contrast, untreated suspensions resulted in four instances of no detection. Experimental results compared with GeneXpert confirm that the detection efficiency achieved with this one-minute preprocessing is comparable to that of conventional 20-minute process. This advancement not only streamlines the diagnostic workflow but also improves the speed and reliability of NTM identification in clinical settings. Our approach offers a significant advancement in the field by enabling direct detection of TB and NTM from raw patient samples within 90 minutes. This represents a major improvement over traditional culture methods, which typically require at least ten days or more. The *fastNTM* technology not only accelerates the diagnostic process but also enhances sensitivity and specificity, as demonstrated.

In conclusion, the multiplex detection capability of *fastNTM* enables the simultaneous identification of multiple targets in a single reaction. This feature is especially advantageous in clinical settings, where rapid and accurate diagnosis is essential for effective patient management. The ability to quickly detect MTB and NTM empowers physicians to make critical decisions regarding patient care and therapy during a single medical encounter, thereby enhancing the efficiency and efficacy of treatment.

## Data Availability

Data available on request due to ethical restrictions.
