# Supplementary material for: Development and evaluation of a multiplex molecular point-of-care assay for direct identification of Mycobacterium tuberculosis and prioritized non-tuberculous mycobacteria
Source: Front Cell Infect Microbiol. 2025 Aug 13;15:1560870. doi: 10.3389/fcimb.2025.1560870 (PMC12380709; doi:10.3389/fcimb.2025.1560870)
Supplement: Supplementary file 1 [file Table1.docx]

Supporting Information for

**Development and evaluation of a multiplex molecular point-of-care assay for direct identification of *Mycobacterium tuberculosis* and prioritized non-tuberculous Mycobacteria**

**Qiao-Lian Yi, Yun Wu, et al.**

**Corresponding author: Qi-Wen Yang and Ying-Chun Xu**

The file includes:

Figure S1. Clinical workflow of positive mycobacterial cultures confirmation.

Table S1. Oligonucleotide sequences for fastNTM to detect target bacteria.

Table S2. The limit of detection of each species.

Other Supplementary Material for this manuscript includes the following are available from the corresponding author with reasonable request.

**Table S1** Oligonucleotide sequences for *fastNTM* to detect target bacteria.

| **Channel** | **Detected target** | **Oligonucleotide sequences** | **Tm** |
| --- | --- | --- | --- |
| channel 1 | *M. gordonae* | Primer-F: CGTGAGGGGTCATCGTCTGT | 69.1-75.0 |
|  |  | Primer-R: ACGACAACGCATACATTTTGAT |  |
|  |  | Probe: AGGCAACACCCTCGGGTGCTGTCCCGCCATCTT |  |
| channel 1 | *M. scrofulaceum* | Primer-F: GGCCCTGAGGCAACACT | 65.0-69.0 |
|  |  | Primer-R: ATCCGTTTAGATGCTCGCAACC |  |
|  |  | Probe: CTGAGTGGTGTCCCTCCATCTTGGTG |  |
| channel 2 | *M. fortuitum* | Primer-F: CACACTATTGGGCTTTGAGAC | 73.0-78.0 |
|  |  | Primer-R: TGCTAGATGCTCGCAACCACT |  |
|  |  | Probe: TGTCGGCGTGTTGTTGCCTCACTTTGGTGGT |  |
| channel 3 | *M. abscessus* | Primer-F: TGTCACCCTGCTTGGTGGT | 65.0-76.0 |
|  |  | Primer-R: CACCATGCGCCCTTAAGAACTT |  |
|  |  | Probe: CGAGCGAGGCTATGTTTAGATGCTCGCAACCAC |  |
| channel 4 | *M. avium* | Primer-F: GGGTGCGCAACAGCAAAT | 63.0-70.0 |
|  |  | Primer-R: CCACTATCCAATACTCAAACACCAC |  |
|  |  | Probe: CCCTGAGACAACACTCGGTCCGTCCGTGTGG |  |
| channel 4 | *M. intracellulare* | Primer-F: GGGTGCGCAACAGCAAAT | 70.1-76.0 |
|  |  | Primer-R: CCACTATCCAATACTCAAACACCAC |  |
|  |  | Probe: CCCTGAGACAACACTCGGTCCGTCCGTGTGG |  |
| channel 5 | *Bacillus subtilis* (Internal control) | Primer-F: GGACCTGTCGTTGACGTACG | 79.0-82.0 |
|  |  | Primer-R: AAGTGCGACCTCTAGCGTT |  |
|  |  | Probe: CACAACCAGCAACAGGTGAAAATGAAGTAGGC |  |
| channel 5 | *M. kansasii* | Primer-F: GTAGTGGACGAAAGCCGGG | 66.0-73.0 |
|  |  | Primer-R: CGTAGGGCAACGCATCCAT |  |
|  |  | Probe: TCTGTTCGAGAGTTGTCCCACCATCTTGGTGG |  |
| channel 6 | *M. marinum/ulcerans* | Primer-F: AAGCCGGGTGCACAACAAC | 66.0-78.0 |
|  |  | Primer-R: AAAGGCAGCGCATCCAATT |  |
|  |  | Probe: CTGAGGCAACATCTCTGTTGGTTTCGGGAT |  |
| channel 7 | MTBC | Primer-F1: CATCGACCTACTACGACCACAT |  |
|  |  | Primer-R1: GGGCACCGTAAACACCGT |  |
|  |  | Probe-1: CGCGAGCTGCGCGATGGCGAACT |  |
|  |  | Primer-F2: GACGAAGCCGTAGAGGCG |  |
|  |  | Primer-R2: CTTGAGCACCAGGGCGT |  |
|  |  | Probe-2: CCCGCTCGATGCCGGCCCGTATA |  |

**Materials and Methods**

**Clinical workflow of positive mycobacterial cultures confirmation**

The original clinical samples for the mycobacterial culture had been cultured at 37℃ using the BD BACTEC MGIT960 system (Becton Dickinson, Franklin Lakes, NJ, USA). Once a sample displaying positive results in the MGIT system, smears of subsequent Auramine O fluorescence staining and Ziehl-Neelsen acid-fast staining were performed to exclude false-positive results. MGIT960-positive cultures with negative staining results were then sub-cultured aerobically on Löwenstein-Jensen medium at 37℃. In this study, 43 cultures were involved MGIT-positive but staining-negative. Upon obtaining positive results from the sub-culture, additional Auramine O staining and Ziehl-Neelsen staining were performed again to ensure the growth of suspected-mycobacteria. Then conduction of NTM was determined by Tuberculosis and Non-Tuberculous Mycobacteria Real-time PCR Detection Kit (CapitalBio Technology Inc., Beijing, China).

**
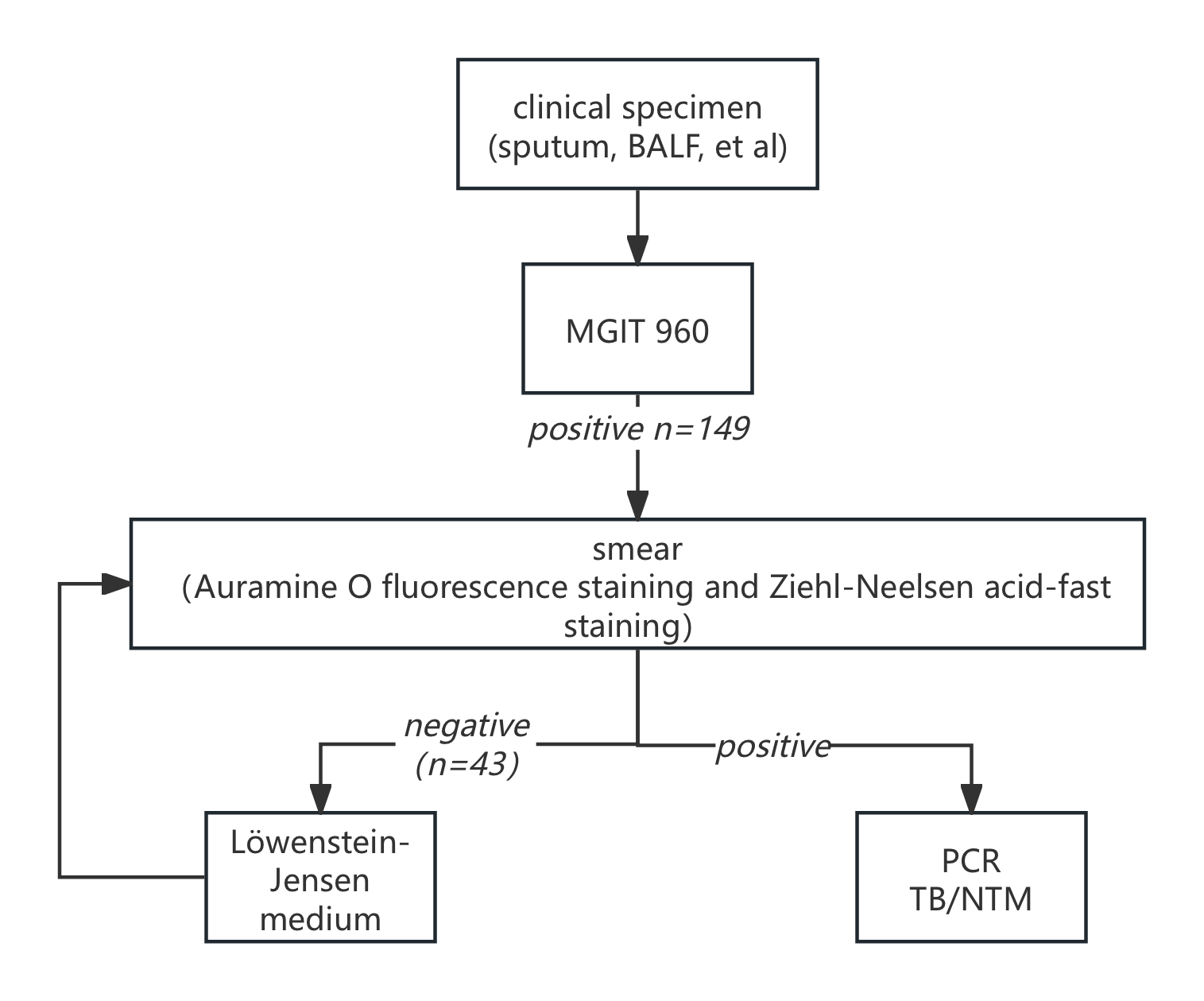
**

**Figure S1** Clinical workflow of positive mycobacterial cultures confirmation. All samples were confirmed NTM by Tuberculosis and Non-Tuberculous Mycobacteria Real-time PCR Detection Kit.

**Table S2 The limit of detection of each species**

| Species (*n* = 10) | target ^a^ (mean±SD, CV%) | IC ^b^ for Ct (mean±SD, CV%) | IC for Tm (mean±SD, CV%) |
| --- | --- | --- | --- |
| *M. tuberculosis* | 39.25 ±0.45, 1.14% | 32.36 ±0.17, 0.52% | 79.86 ±0.48, 0.61% |
| *M. avium* | 68.69 ±0.26, 0.38% | 32.57 ±0.58, 1.77% | 80.35 ±0.78, 0.97% |
| *M. intracellulare* | 73.96 ±0.31, 0.41% | 32.59 ±0.53, 1.62% | 80.09 ±0.68, 0.85% |
| *M. abscessus* | 73.60 ±0.33, 0.44% | 32.77 ±0.36, 1.11% | 79.99 ±0.50, 0.63% |
| *M. kansasii* | 68.86 ±0.46, 0.67% | 32.58 ±0.23, 0.70% | 79.95 ±0.55, 0.69% |
| *M. fortuitum* | 74.73 ±0.23, 0.31% | 32.54 ±0.51, 1.56% | 80.25 ±0.40, 0.50% |
| *M. marinum* | 68.74 ±0.33, 0.48% | 32.63 ±0.56, 1.72% | 80.19 ±0.59, 0.74% |
| *M. gordonae* | 73.96 ±0.34, 0.46% | 32.62 ±0.53, 1.64% | 80.19 ±0.58, 0.73% |
| *M. scrofulaceum* | 68.14 ±0.35, 0.51% | 32.98 ±0.26, 0.78% | 80.08 ±0.58, 0.73% |
| NC | NA | 32.66 ±0.33, 1.02% | 80.06 ±0.50, 0.62% |

^a^ Indicator for MTBC and NTM are Ct and Tm respectively.

^b^ IC: internal control; NC: negative control, negative sputum; NA: not applicable.
